# Bottleneck Genes and Community Structure in the Cell Cycle Network of S. pombe

**DOI:** 10.1371/journal.pcbi.0030103

**Published:** 2007-06-01

**Authors:** Cécile Caretta-Cartozo, Paolo De Los Rios, Francesco Piazza, Pietro Liò

**Affiliations:** 1 Laboratoire de Biophysique Statistique, Ecole Polytechnique Fédérale de Lausanne, Lausanne, Switzerland; 2 Computer Laboratory, University of Cambridge, Cambridge, United Kingdom; The University of Tokyo, Japan

## Abstract

The identification of cell cycle–related genes is still a difficult task, even for organisms with relatively few genes such as the fission yeast. Several gene expression studies have been published on S. pombe showing similarities but also discrepancies in their results. We introduce a network in which the weight of each link is a function of the phase difference between the expression peaks of two genes. The analysis of the stability of the clustering through the computation of an entropy parameter reveals a structure made of four clusters, the first one corresponding to a robustly connected M–G1 component, the second to genes in the S phase, and the third and fourth to two G2 components. They are separated by bottleneck structures that appear to correspond to cell cycle checkpoints. We identify a number of genes that are located on these bottlenecks. They represent a novel group of cell cycle regulatory genes. They all show interesting functions, and they are supposed to be involved in the regulation of the transition from one phase to the next. We therefore present a comparison of the available studies on the fission yeast cell cycle and a general statistical bioinformatics methodology to find bottlenecks and gene community structures based on recent developments in network theory.

## Author Summary

Because of the diversity in technological and analytical approaches, published microarray studies on a given organism show similarities as well as differences. While a great amount of data is now available, there is a general need for comprehensive methodologies that would allow us to analyze and compare all these data. We propose a general statistical bioinformatics approach based on recent developments in network theory, and we present an application to three different cell cycle–regulated genes datasets on the fission yeast. We introduce the periodic cell cycle network built upon microarray data on gene expression, and we study the properties and the stability of its community structure. We show that the periodic cell cycle network of the fission yeast is characterized by four clusters separated by bottleneck structures corresponding to cell cycle checkpoints. We identify a set of genes located on these bottlenecks, and we propose them as potential new cell cycle regulators involved in the control of the transition from one phase to the next. Our approach can be applied to other similar complementary datasets or to any gene expression datasets to reveal the community structure of the corresponding network and to isolate genes potentially involved in cell cycle regulation.

## Introduction

The cell cycle is a highly controlled ordered set of events, culminating in cell division into two daughter cells. The cell division requires doubling of the genome (DNA) during the synthesis phase (S phase) and halving of that genome during mitosis (M phase). The period between M and S is called G1; that between S and M is G2. Microarray technologies have been used to identify cell cycle genes in several organisms (human, *Saccharomyces cerevisiae, Schizosaccharomyces pombe,* and Arabidopsis thaliana) [1,2]. Datasets are generated using different synchronization conditions and time measurements [3]. Among them, centrifugal elutriation produces a homogeneous population of small cells early in their cell cycle, while temperature-sensitive mutants show arrest in specific cell cycle stages at a restrictive temperature. The mRNA is extracted at a number of time points following synchronization. After measuring the expression level for all genes, those expressed in a periodic manner are identified using several different methods, such as Fourier analysis [4,5]. The result is the assignment of a cell cycle phase to each gene that has been detected as periodically regulated.

The cell cycle of the fission yeast S. pombe lasts approximately 3 h. Its structure is the same as in all other eukaryotes. However, S. pombe is the only yeast that divides by fission, a symmetrical process in which the old cell grows until it divides, with the formation of a central mitotic spindle, into two equal new cells. As a consequence, it is characterized by a very long G2 phase of overall increase of the cell mass that covers 70% of the cell cycle. The M phase is marked by chromosome condensation and segregation to opposite ends of the cell. Then the cell goes rapidly through the G1 phase with the synthesis and accumulation of active proteins required for DNA replication. Therefore, by the time cytokinesis occurs, the S phase is completed and an entire complement of chromosomal DNA is synthesized.

Recently, three independent studies have made available gene expression data on the cell cycle of fission yeast [6–8]. They measured gene expression as a function of time in both wild-type elutriation and cdc25 block-and-release experiments, and they identified different datasets (Table 1). A total number of almost 1,400 genes are found to oscillate in the three studies. About 10% of these genes are identified as periodically regulated in all the three studies and less than 30% in at least two of them. The definition of cell cycle–regulated genes is far from being rigorous. The identity and the numbers of genes in the periodic datasets strongly depend on the approach and on how conservative one wants to be. Instead of looking at the single gene, we define a periodic cell cycle network and study its cluster structure to find universal properties that are stable despite differences in the datasets. Both Rustici et al. [6] and Peng et al. [7] identified four clusters of periodic genes, corresponding roughly to the four main phases of the cell cycle, while Oliva et al. [8] proposed eight different clusters. Nevertheless, the distribution of the phases only reveals two clear expression waves. We consider the periodic cell cycle network corresponding to the intersection of the three datasets, and we study the clustering and its stability [9,10]. At first, two main components appear. The first one groups all genes in the M, G1, and S phases, and the second corresponds to the entire G2 phase. They fit the pattern shown in the distribution of the phases. Further search for hierarchical substructures of these two clusters shows that the M and G1 phases form a robustly connected single component, while the G2 phase can be divided into two clusters, and the S phase forms a separated component of its own. The stability measure indicates that a structure made of four clusters represents the more reliable pattern of the distribution of the periodic genes on the cell cycle. These clusters are separated by bottleneck structures corresponding to cell cycle checkpoints. We will discuss a set of genes located on these bottlenecks.

**Table 1:**
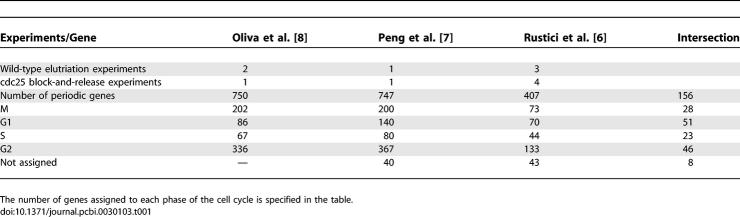
Number of Wild-Type Elutriation and cdc25 Block-and-Release Experiments and Number of Genes Identified as Periodically Regulated in the Three Studies and in Their Intersection

## Methods

### Datasets and Quality Assessment

Genome-wide microarray expression data as well as a list of periodically regulated genes from each study are available online, along with phase and amplitude values assigned to each gene [11,12]. We considered data obtained from elutriation experiments in the three studies [4].

We analyzed the distribution of the phases and amplitudes in each periodic set. We then considered the distribution of phase differences as a more reliable comparative parameter among the three studies. After having studied the histograms, we made use of kernel density plots to remove insignificant bumps and reveal real peaks. Histograms strongly depend on the choice of the bin grid and on the starting point. Kernel density estimators are smoother than histograms and converge faster to the true density [13–15]. The choice of a proper bandwidth is still an important issue, and it should represent a compromise between smoothing enough and not smoothing too much to smear real peaks away. We computed the histograms averaging over a large number of shifts of starting points and considering very small bins with data-dependent bandwidth. Changes in the bandwidth do not affect our qualitative analysis.

The three periodic datasets show differences in size. Searching for an explanation for this discrepancy, we computed the cyclic Fourier component obtained by the time series of each gene in the genome. We then compared it with the one obtained from randomly reshuffled expression data to generate a *p*-value for the periodicity of the corresponding gene. This indicator represents the probability that the observed oscillation occurs by chance. The smallest *p*-values correspond to the most cyclic genes. The amplitude of the oscillation also contributes to the *p*-value in such a way that genes with greater amplitude have a smaller *p*-value. We studied the normalized distribution *P*(*p*) of the *p*-values for the three studies.

### Clustering and Entropy Measure

We defined a network represented by a complete graph (each node is connected to all other nodes in the graph) where each node corresponds to a gene whose expression was identified as periodically regulated during the cell cycle in the corresponding study. In all datasets, a gene is assigned a phase *ϕ_i_* and an amplitude *A_i_* at the expression peak. The most useful parameter for comparison is the phase difference, a measure of the expression peaks distance between genes in the cell cycle. The link between node *i* and node *j* is thus assigned a weight *ω_ij_* given by the expression

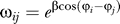
where *ϕ_i_* is the phase of node *i* and *β* is a tuning parameter. We studied the degree distribution and clustering coefficient of the resulting network. As we are dealing with a complete, weighted graph, we considered appropriate definitions of the *weighted degree* (strength) and of the *weighted clustering coefficient* [16]. We also considered the binary network obtained by fixing a threshold *t* and keeping only links with *ω_ij_ ≤ t*. We studied the degree distribution and the correlation between the degree and the phase for each gene.

We applied the Markov clustering algorithm (MCL) [17,18] to study the cluster structure of the periodic cell cycle network. Unlike most clustering algorithms, the MCL does not require the number of expected clusters to be specified beforehand (this condition can be very limiting and time-consuming when there is no specific a priori information regarding the network structure), and it can easily identify possible hierarchies of substructures. Note that the MCL has been used in approaching several bioinformatics classification problems [19–22]. The basic idea underlying the algorithm is that dense clusters correspond to regions with a larger number of paths. A random walk has a higher probability to stay inside the cluster than to leave it soon. The crucial point lies in deliberately boosting this effect by an iterative alternation of expansion and inflation steps. The algorithm iterates three steps. Given a network with *n* vertexes, it takes the corresponding *n* × *n* adjacency matrix *A* and normalizes each column to obtain a stochastic matrix *M*. It takes the *k* ^th^ power *M^k^* of this matrix (expansion) and then the *r* ^th^ power *m_ij_^r^* of every element (inflation).

In the case of a weighted graph, such as the periodic cell cycle network, the probability of the random walk is proportional to the weight of the link. In our analysis, the expansion parameter *k* is always taken equal to 2, while the granularity of the clustering is controlled by tuning the inflation parameter *r*. In addition to the parameter *r*, we also introduced a control parameter *β*. This parameter allows us to speed up the process. In a first analysis with *β* = 1, the algorithm needed high values of *r* to identify the first two clusters and then went on very slowly. This behavior can be explained by the fact that our periodic cell cycle network is a complete graph. In what follows we will always consider *β* = 10.

To study the robustness of the results given by the MCL, we considered the stability of the clustering as related to the identification of unstable nodes [23]. A node is unstable if it typically lies at the borders of different clusters, so that the algorithm has some difficulty in assigning it to either of its basins of attraction. To measure the stability of the clustering patterns, we added random noise on the weights of all links in the network and studied the clustering after many realizations. Let *P_ij_* denote the probability that the link between node *i* and node *j* connects two nodes inside the same cluster (*P* is equal to 1 for a link that is always kept and 0 for a link that is always cut by the algorithm). By fixing a threshold *θ* (typically *θ* = 0.8) and eliminating all the links with *P_ij_* ≤ *θ*, we obtain a certain number (greater than or equal to the number of original clusters) of disconnected components. Nodes belonging to small components that cannot be identified with any of the original clusters can be defined as unstable. Figure 1 shows a very simple network with a cluster structure made of three components. Through different random noise realizations, the green node is alternatively assigned to either of its basins of attraction. The resulting probabilities *P_ij_* of the two links that connect the node to the rest of the network are ≤ 0.8. The node is thus identified as a single component that does not correspond to any cluster, and it is defined as unstable. In the case of the periodic cell cycle network, the weights on the links are modified as *ω_ij_* (1 + Δ*_ij_*) where Δ*_ij_* are Gaussian deviates with 0 mean and standard deviation 0.5. Results do not change if we increase the noise strength.

**Figure 1:**
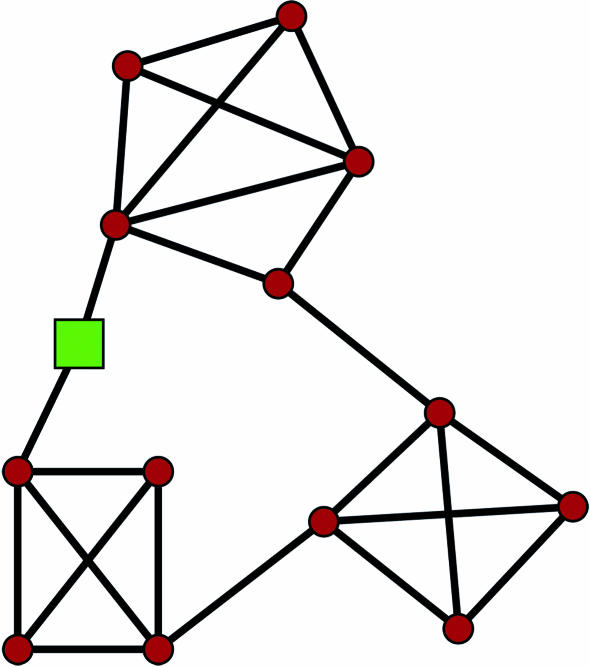
Simple Graph Made of Three Components The MCL is able to identify the three clusters. The analysis of the instability of the clustering described in Clustering and Entropy Measure identifies the node coloured in green as unstable.

Using the probabilities *P_ij_*, we introduce the average *clustering entropy* per edge:

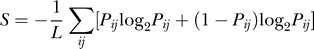
The sum is over all edges, and the entropy is normalized by the total number of edges *L*. If the network is totally unstable (*P_ij_* = ½ for all edges), then *S* = 1; if the network is perfectly stable (*P_ij_* = 1 or 0 for all edges), then *S* = 0. In the case of the MCL algorithm (and of any other clustering algorithm defined through a parameter), we can either consider single values of the function *S* at fixed values of *r*, or we can study the landscape of the clustering entropy as a function of the clustering parameter.

### Overlap and Agreement

To compare the results given by the different studies and to analyze the structure of the cell cycle given by a more reliable core of periodic genes, we studied the intersection of the three datasets.

We observed that even if a gene is identified as periodically regulated by the three studies, the assigned phase values *ϕ_i_* can be substantially different. We considered a distance matrix *A* whose elements *a_ij_* are given by the phase difference *ϕ_i_* − *ϕ_j_* between the expression peaks of genes *i* and *j* (a symmetric matrix with all zeros on the diagonal). Having three distance matrices, one for each dataset (only genes in the intersection of the three experiments are considered, in order to obtain three matrices of the same size), we applied the Mantel test, which computes a correlation between two *n* × *n* distance or similarity matrices. It is based on the normalized cross-product:

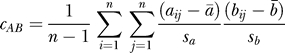
where *a_ij_* and *b_ij_* are the generic elements of the two matrices *A* and *B* we want to compare, *ā* and *b̄* are the corresponding mean values, and *s_a_* and *s_b_* the standard deviations. The null hypothesis (NH) is that the observed correlation between the two distance matrices could have been obtained by any random arrangement. The significance is evaluated via permutation procedures. The rows and columns of one of the two matrices are randomly rearranged, and the resulting correlation is compared with the observed one.

We also computed a general error on the phase values as the distance between two successive points of the time series, assuming that inside the interval between them it is impossible to precisely assign the phase value. According to this error, we studied the agreement on the phase values.

After this preliminary analysis on the agreement of the three datasets, we considered the network corresponding to the intersection. We studied the clustering and its stability. The computation of the strength of the nodes (the sum of the weights of all links of a node) allows us to identify genes that are located at the borders of the clusters [16]. Their strength is significantly smaller than the mean value of the network.

## Results

### Exploratory Data Analysis

As a preliminary comparative study of the different datasets, we analyzed the distributions of the phase and amplitude of the periodic genes in the three datasets. The amplitude distribution is very similar across the studies and well fitted by a bell-shaped distribution. It does not give further information on data. The phase distribution is more interesting (Figure 2, left). The overall behavior is universal, with two main peaks separated by low-expression regions. The first one corresponds to the transition from phase S to phase G2 and the second from phase G2 to phase M. We also introduced the phase distribution of the set of ~800 genes identified as periodic in the budding yeast S. cerevisiae [24] for comparison. It shows a single expression wave corresponding to the S and G1 phases of the cell cycle. Discrepancies can be observed in the position and extension of the two peaks across the three studies on S. pombe. Differences in the synchronization technology and phase assignment method are probably at the origin of these deviations. We thus consider the phase difference Δ*ϕ* as a more reliable parameter for comparison. The corresponding distributions are much more similar (Figure 2, right). The common minimum corresponds to the low expression regions between the two peaks in the phase distributions. Slight deviations in the head of the distribution are a consequence of normalization over datasets of different size. Differences in the tail depend on a lack of uniformity in the abundance of genes across the four phases of the cell cycle (Table 1).

**Figure 2:**
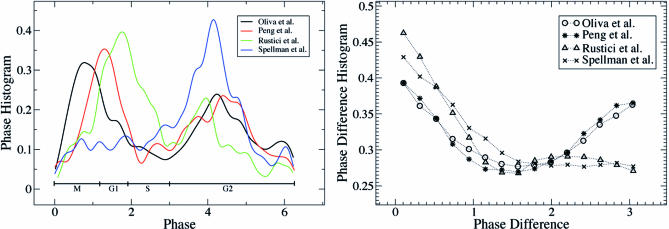
Phase Distributions (Left) and Phase Difference Distributions (Right) for Periodic Genes from the Three Independent Studies on S. pombe and from the Study on S. cerevisiae by Spellman et al. [24] On the *x*-axis, the phase values are given in radians between 0 and 2*π* and the phase differences in radians between 0 and *π*. The *y*-axis always shows the corresponding frequency distribution.

The number of genes identified as periodic in the three studies are quite different. Rustici et al. [6] propose 407 periodically regulated genes, while Peng et al*.* [7] and Oliva et al. [8] indicate bigger sets with ~750 genes each (Table 1). The correct number of periodic genes is still unknown, and whether it is better to focus on a small number or to consider all the results as equally meaningful is an open question. In Figure 3 we show the normalized distributions of *p*-values relative to the cyclic spectral component for all genes from the different studies (time series with two or more consecutive missing data points have been ignored). They depend on time series properties, such as the number of points and intervals that differ from one study to the other. Nevertheless, in all of the three studies, they show inverse power-law behavior. This result tells us that if we consider exclusively the information contained in the time series, there is no characteristic threshold that can be used to separate periodic from nonperiodic genes. In the experiments, the choice of the proper periodic dataset strongly depends on the false discovery rate and on the visual doublecheck of the time series. Some degree of arbitrariness remains in the choice of the cut, and it is co-responsible for the discrepancies between the three studies. The fact that Peng et al. [7] and Oliva et al. [8] presented similar datasets in size is not significant. In their study, Oliva et al. [8] concluded that there is no way to distinguish between periodic and nonperiodic genes. They ranked more than 2,000 genes according to their periodicity indicator, and they finally focused on a set of 750 to establish reasonable comparisons with the other already published datasets on the budding and fission yeasts.

**Figure 3:**
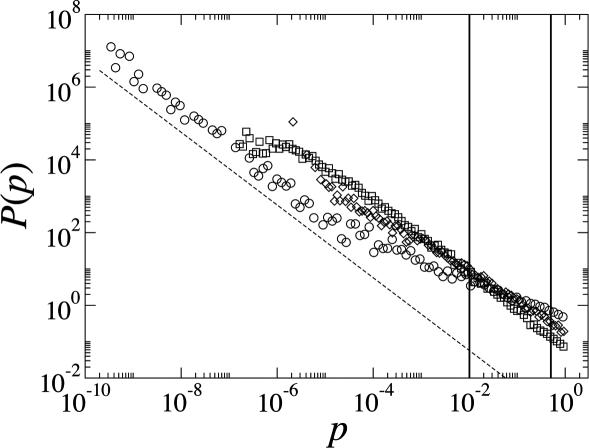
Normalized Distributions of *p*-Values Relative to the Cyclic Spectral Component for All Genes from Three Different Elutriation Experiments Rustici et al. [6] (diamonds), Peng et al. [7] (squares), and Oliva et al. [8] (circles). The dashed line is an inverse power law with exponent −1. The *p*-values have been computed by reshuffling each signal 10^10^ times. 5% and 1% confidence levels are emphasized in the plot.

### Cluster Structure of the Periodic Cell Cycle Network

In Figure 4 we give a graphical representation of the periodic cell cycle network in the three studies. The relevant structure of this kind of network is given by links with higher weight. These links connect genes that are expressed (and probably regulated) at the same time on the cell cycle. Figure 4 shows the binary networks obtained by keeping only links with *ω_ij_* < *t* (in the present case we fixed *t* = 18,000; lower thresholds only affect the thickness of the circular graph). They reflect the time progression of the cell cycle, with the correct sequence of phases. The length of each phase does not correspond to the real extent of the phase in the cell cycle, but rather reflects the corresponding number of periodic genes. The diameter of each node represents the amplitude assigned to the corresponding gene. We observe that in all experiments high-amplitude genes are mostly concentrated in the three shorter phases (M, G1, S). We stress that the threshold *t* was only introduced for graphical purposes and that all analysis were made on the complete, weighted networks.

**Figure 4:**
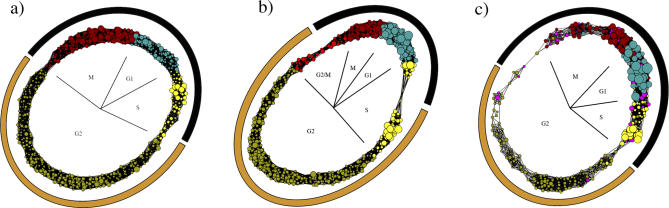
Networks of the Three Sets of Periodic Genes in S. pombe Oliva et al. [8] (A), Peng et al*.* [7] (B), Rustici et al. [6] (C). Different colors identify genes assigned to different phases of the cell cycle. Pink nodes in Rustici et al*.* [6] correspond to genes that have been identified as periodic but not assigned to a specific cell cycle phase. The radius of each node has been set according to the amplitude of the corresponding gene at its expression peak. The black–orange circle provides a visual identification of the two main clusters (the black one and the orange one). This image has been realized with the graph drawing software Visone [30].

The weighted degree distribution highlights that most nodes have high strength, reflecting the completeness and overall uniformity of the network. The correlation between the strength and the phase of each node tells us that genes lying on the low expression regions that separate the two peaks of the phase distribution correspond to nodes with strength significantly smaller than the mean value of the network. Moreover, genes belonging to the M, G1, and S phases (first peak) have greater strength than those belonging to the G2 phase (second peak).

The three networks are characterized by a very high clustering coefficient (*C* ≈ 0.5). Their community structure appears to be robust, as they all split in a relatively small number of groups of nodes (<10) for increasing values of the granularity parameter *r* (Figure 5, top). The progression of the clustering is smooth. At first, all networks are separated into two large clusters (see Figure 4) that the MCL is able to identify at *r* ≈ 1.25. The first one corresponds to the set of genes belonging to M, G1, and S phases of the cell cycle, while the second one collects all genes belonging to the G2 phase. Such clusters reflect the two peaks of the phase distribution (see Figure 2). In all experiments, they appear to be separated by bottleneck structures, which correspond to transitions from one phase to another (more precisely, from phase S to phase G2 and from phase G2 to phase M) and seem to be characterized by the presence of a smaller amount of periodic gene expression (in good agreement with the distribution of gene phases in Figure 2). By increasing the value of the granularity parameter *r*, the two main clusters are respectively split into subclusters, suggesting the presence of a hierarchical organization.

**Figure 5:**
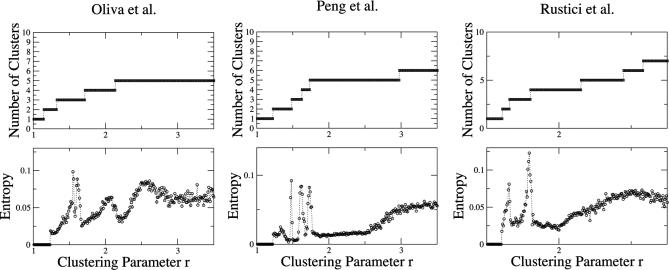
Number of Clusters (Top) and Entropy Landscape (Bottom) as a Function of the Clustering Parameter *r*, always Given on the *x*-Axis Top, the *y*-axis shows the number of clusters identified by the MCL. Bottom, the *y*-axis shows the clustering entropy *S*. A peak in the entropy landscape corresponds to an increase in the number of clusters.

### Stability of Convergence of the MCL

To assess the significance of this scenario, we studied the stability of the cluster patterns in terms of the presence of unstable nodes and of the behavior of the clustering entropy [23]. The cluster structure of the network of periodic genes in S. pombe is highly stable. We identified no more than two or three unstable nodes, depending on the granularity of the clustering. As one might expect, these nodes correspond to genes lying at the bottleneck structures visible in Figure 4 (i.e., genes belonging to periods of transition between different phases in the cell cycle).

Furthermore, there is a correspondence between the number of clusters and the trend of the clustering entropy *S* as a function of the parameter *r* (Figure 5). The jump from a partitioning level to the following shows up as a peak in the entropy landscape. In Figure 5 (top) we see that the number of clusters sometimes remains constant for a large interval of values of the parameter. In these cases, increasing *r* from the first-cut value (actually corresponding to a peak in the entropy) results in a decrease of the entropy until it reaches a minimum. This minimum represents a more stable configuration of the clustering. This picture holds until the entropy reaches saturation.

### Gene Network of the Intersection

In each of the three studies, all genes in the genome were ranked according to a periodicity indicator. Comparing the three ranked lists, it is possible to observe that the agreement between the three datasets is much stronger between top-ranked genes, which means genes that are found to be more strongly regulated [8]. It is therefore interesting to study the minimal list given by the intersection of the three datasets. It comprises only genes that have been identified as periodic by all groups and that are thus placed on top of the three ranked lists.

The intersection is given by a set of 156 genes, that is, about 10% of the entire pool. The Mantel test returns a value *c_AB_* ≈ 0.8, showing a good correlation between the distance matrices of the three experiments. To assess the statistical significance of this result, we compared it with the NH. We obtained a *p*-value that scales as *p*(*n*) = e^−*n*^ in which *n* is the number of pairwise rearrangements of the rows and columns (calculated over 10^5^ randomizations). This means that the mere reshuffling of 20% of the matrix gives *p* ~ e^−10^. The actual value is thus significant against the NH.

The distribution of the number of genes on the four phases of the cell cycle is now different (Table 1). Less then one-third of the shared genes belong to the G2 phase, with most genes belonging to the M–G1–S cluster, and, more precisely, half of them to the G1 phase. We found that even if a gene is periodic in more than one group, the corresponding phase values can be different. The best agreement between the phase values is for genes in the M–G1–S cluster. Less than 20% of the genes show an agreement of the three phase values within the error, while ~60% of the genes show good agreement at least between two values.

The clustering structure of the intersection shows the same two main clusters as in the separated networks (Figure 6). Further analysis of the hierarchical substructure reveals that genes in the M and G1 phases are strongly connected and cannot be separated, while the S phase forms an independent component. Moreover, the G2 phase shows at least two separated subclusters. The resulting four clusters are separated by bottleneck structures. Two of them correspond to the phase transitions already observed in the networks of the entire datasets. The third one is less evident, and it corresponds to the transition between the G1 and S phases. This clustering pattern is very robust. No more than two or three nodes can be identified as unstable, and they are located on the G1–S bottleneck. We computed the strength of the nodes to identify genes that lay at the borders of the clusters. A list of these genes and their functions is shown in Table 2.

**Figure 6:**
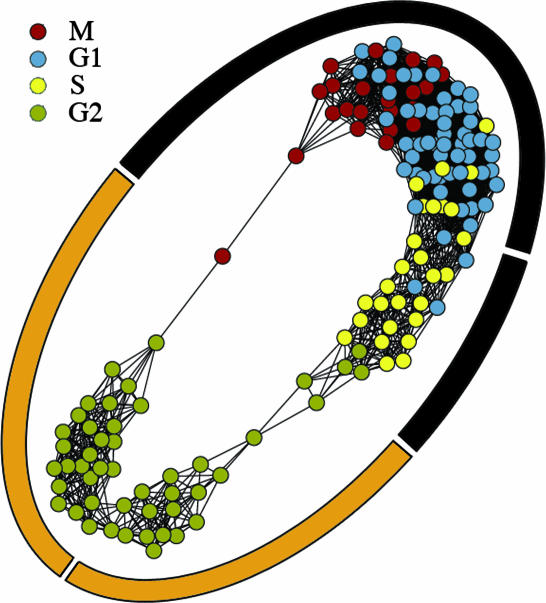
Network of 156 Genes Identified as Periodically Regulated in the Three Studies (~10% of the Entire Pool) The black–orange circle provides a visual identification of the two main clusters (the black one and the orange one) and of the relative hierarchical subclusters. This image has been realized with the graph drawing software Visone [30].

**Table 2:**
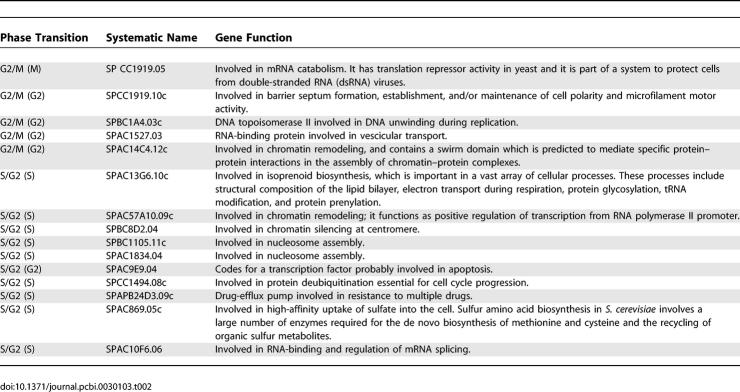
Genes Located at the Bottlenecks That Separate the Two Main Clusters in the Network of the Intersection of the Three Datasets (the Term in Brackets in Column 1 Is the Assigned Phase)

## Discussion

We have carried out an extensive comparative analysis of the results from three independent groups working on cell cycle data. The power-law distribution of the *p*-values (Figure 3) in the three datasets shows that a large number of genes are above the 1% and 5% confidence levels. There is no apparent change in the exponent of the power law in these regions. This implies that the information contained in the time series is not enough to establish a clear division between strictly periodic and nonperiodic genes. It is now known that the cell cycle is central to a large number of subnetworks dedicated to other cell activities. Disruptions in pathways leading to DNA repair, signaling, membrane lipid and protein formation, and protein degradation may affect the survival potentialities of the daughter cells. This argument suggests that a large number of genes may be loosely involved in the cell cycle even if they are not main players of it. The numbers and identity of genes in the periodic sets thus strongly depend on false discovery rate and interpretation of the data [25]. At first, a statistical method such as Fourier analysis is applied in a rather blind way. Then, cell cycle profiles are filtered for minimal amplitude and doublechecked by eye. The visual inspection of the gene expression profiles has its advantages, but obviously a different reproducibility from that of a statistical analysis. Different methods may extract different types of information and may be difficult to compare. The network defined in Clustering and Entropy Measure provides a representation of the entire periodic cell cycle with no need to focus on the single genes or phase values. Figure 4 shows that, despite the differences in the datasets, some properties of the general structure of the network are universal across the three studies.

Although G2 is the longest phase in the cell cycle, it is not the most densely populated. It spreads over ~70% of the cell cycle, but in all studies, it contains no more than a half of the identified periodic genes (see Table 1). This is not unexpected. In *S. pombe,* cell division occurs at the end of S phase, implying that the G2 phase represents a long period of mass increment. Most genes are expressed during the entire process and thus do not have a defined expression phase. Some genes, which are probably involved in more specific tasks, are periodically regulated. We studied the amplitude of each gene at the expression peak as a function of the corresponding phase, and we observed that genes are more strongly regulated during the M, G1, and S phases than in the G2 phase. The study of the intersection of the three datasets shows that genes and phase agreement is not so good on the G2 phase. This suggests that G2-phase genes are more likely to be false positives and probably represent an overestimation of the number of genes that are truly periodic. Moreover, in Rustici et al. [6] (dataset with 407 periodic genes) G2-phase periodic genes represent a little more than one-third of the entire pool, while in the other experiments (both showing a much bigger pool of ~750 periodic genes), the G2-phase genes correspond to about half the dataset (Table 1).

The distributions of the phase differences in the three datasets are very similar, and they clearly identify two waves of periodic expression (Figure 2). They are peaked in the M–G1–S phases and the G2 phase, respectively, and they are separated by short, quiet periods in which very few periodic genes are expressed. Nevertheless, both Rustici et al. [6] and Peng et al. [7] identified four clusters of periodic genes, roughly corresponding to the four main phases of the cell cycle, while Oliva et al. [8] proposed eight different clusters. This clustering is not consistent with the distribution of periodic genes on the cell cycle. To study the topological clustering of the cell cycle, we applied the MCL to the periodic cell cycle network. In all studies, the first hierarchical level of the clustering shows two main clusters, one corresponding to the M–G1–S phases and the other to the G2 phase, separated by bottleneck structures with very few genes. This pattern reflects the behavior of the phase distribution. The study of the lower hierarchical levels reveals that genes in the M and G1 phase form a strongly connected cluster that cannot be further divided by the algorithm. On the other hand, genes in the S phase are grouped in an independent component, and the G2 phase can be partitioned into at least two clusters. This pattern is universal across the different studies, suggesting that the topology of the network contains information on the biological processes involved in the cell cycle.

The stability of the clustering shows the robustness of the structure against the presence of false positives and false negatives in the datasets. The entropy landscape does not change much across the experiments. It always reaches saturation when the network splits into six or seven clusters. In Figure 5, we see that Peng et al. [7] and Rustici et al. [6] show a common stable minimum of the entropy at *r* ≈ 2. In both studies, this minimum corresponds to a separation into five clusters. A similar result holds for Oliva et al. [8] with a slightly different value of the clustering parameter (r ≈ 2.2). In the network of the intersection, the entropy landscape shows a minimum that corresponds to a separation into four or five clusters. We thus suggest that a basic structure made up of four clusters (with eventually a fifth one in the G2 phase) could be the most reliable picture of the clustering pattern. Genes in the M and G1 phases form the first component, genes in the S phase form the second component, and then genes in the G2 phase form the last two components.

For the sake of comparison, we also applied a different clustering algorithm to the periodic cell cycle network. In recent years, several methods have been proposed to reveal the community structure of very heterogeneous networks. Among them, only a few can successfully handle a complete and weighted graph. One possible choice is an algorithm based on modularity (*M*) optimization (a measure of the difference between the number of links inside a given module and the expected value for a randomized graph of the same size and degree distribution) [26]. We considered a recent method based on simulated annealing to obtain clustering by direct maximization of *M* [27]. The results are very similar to the more reliable picture obtained by the MCL (as described in the previous paragraph). The application to the periodic cell cycle network in Rustici et al. [6] and Oliva et al. [8] returns a structure made of four clusters: one corresponding to the M phase and part of the G1 phase, one corresponding to the end of the G1 phase and the S phase, and two modules inside the G2 phase. In the case of Peng et al. [7], a fifth cluster corresponding to the G2–M phase is identified. The bottleneck structures identified by the MCL are well respected. The main difference seems to be the partitioning of genes in the G1 phase between the two clusters corresponding, respectively, to the M phase and the S phase. To explain this behavior, we refer to a recent work on resolution limits in community detection [28]. The authors give evidence that modularity optimization may fail to identify modules smaller than a certain scale, depending on the total number of links in the network and on the number of connections between the clusters. More precisely, even a module whose size is on the order of the size of the entire network may not be resolved if it has a number of external links on the order of the number of connections inside the module itself. In the exploratory data analysis we showed that only the two main communities of the periodic cell cycle network are revealed by the phase distribution. However, the MCL is able to identify a cluster substructure. Our discussion of the results points out that the module corresponding to the S phase is the last one isolated by the algorithm, and that the bottleneck between the G1 and S phases is the less evident and more unstable one. The number of links connecting this module to the M–G1 cluster is on the order of the number of internal links. According to these arguments, the modularity algorithm would rather split the big M–G1–S cluster into two symmetric subclusters than separate the smaller S phase from the larger M–G1 component.

The analysis of the stability of the clustering in the network of the intersection reveals its robustness. There are no unstable nodes on the two main bottlenecks, the G2–M and the S–G2, and only one or two unstable nodes on the border between the G1 and S phases and between the two clusters in the G2 phase. These results confirm the significance of these structures and their role in the biology of the cell cycle. The bottlenecks are strongly correlated to cell cycle checkpoints. These are cellular pathways, induced by DNA damage, that block cell cycle progression or slow the rate at which the phase proceeds. According to the cell cycle stages, there are at least three DNA damage checkpoints: G1–S (G1) checkpoint, intra–S phase checkpoint, and G2–M checkpoint. We thus investigated those genes that are located at the borders between different clusters that correspond to cell cycle checkpoints. We ranked the nodes according to their strength, starting from the one with the smallest value, and we kept those that are on top of the ranking in at least two datasets. These nodes represent genes that are located on the bottlenecks corresponding to cell cycle checkpoints. This means that they are periodically expressed during the transition from one phase to the next. A list of these genes and their functions is shown in Table 2. Most of them have important functions, and we propose them as potential new cell cycle regulators involved in the control of the transition from one phase to the next [29].

The approach described in this paper is an example of comparative analysis and can be applied to other, similar complementary datasets. Moreover, the periodic cell cycle network can be built from any gene expression dataset. The study of the clustering and the stability measure reveal the more reliable community structure of this network. The identification of nodes lying at the borders of different clusters can contribute to the isolation of genes potentially involved in cell cycle regulation. As a future development, we will consider applying this method to gene expression data on the human cell.

## Abbreviations

MCL: Markov clustering algorithm
